# Tool for Nursing Acuity Measurement - Swedish version (NAM-S) for somatic in-patient care: development, validity, and reliability

**DOI:** 10.1186/s12913-026-14036-w

**Published:** 2026-01-27

**Authors:** Kristin Dellesjö, Maria Engström, Annica Björkman, Karin Myrberg

**Affiliations:** 1 Faculty of Health and Occupational Studies, Department of Caring Sciences, University of Gävle, Gävle, Sweden; 2 Centre for Research and Development, Region Gävleborg/Uppsala University, Uppsala, Sweden

**Keywords:** Nursing acuity, Nursing intensity, Nursing workload, Patient acuity, Patient classification, Psychometric properties, Reliability, Somatic care, Tool development, Validity

## Abstract

**Background:**

Nursing acuity measurements refer to the assessments of the intensity of nursing care required by a patient, helping to determine appropriate levels of nursing resources and staffing requirements. The aim of this paper is to describe the development and validation of a tool for measuring nursing acuity within adult somatic in-patient care settings, distinguishing between advanced nursing tasks and basic nursing tasks.

**Method:**

The nursing acuity tool was developed through an iterative process in the context of a Swedish healthcare region. The methodological process involved a literature search, exploration of non-validated tools used within Swedish healthcare, and nursing staff discussions to reach a conceptual tool. This tool underwent pilot testing across five wards, as well as testing for content- and construct validity, and inter-rater reliability.

**Results:**

After an iterative process of development and testing, we reached a final version of a tool, subsequently named the Swedish Nursing Acuity Measurement (NAM-S). The results demonstrated acceptable validity and inter-rater reliability.

**Conclusions:**

Our study provided support that the NAM-S can be used for assessing nursing acuity within adult somatic in-patient care. To refine the model, further examinations of its usefulness and experiences of the practical use of NAM-S are warranted.

**Supplementary Information:**

The online version contains supplementary material available at 10.1186/s12913-026-14036-w.

## Background

Adequate nurse staffing is essential to the quality of care. Assessing the optimal number and type of nursing staff necessary to meet patient care requirements has long been regarded as a fundamental aspect of healthcare management [1, 2]. Nursing workforce shortages, increasing demand for care, and an aging population are emerging challenges to maintaining this balance [3–6]. Traditionally, there has been much focus on supply and less on demand [7], although high workloads are clearly associated with nurse turnover and intensions to leave the profession [8, 9]. Consequently, the need for accurate data on patient care requirements has steadily increased.

A traditional approach to staffing is the nurse-to-patient ratio [10]. This method has been debated due to the variability and unpredictability of patient needs, as patients with the same medical condition in the same ward may have varying requirements for both basic and advanced nursing care [11]. Hence, an emerging strategy for accurately assessing nurse staffing levels involves linking nursing staff and assignments to the classification of each patient’s care needs [12]. This approach is commonly known as nursing acuity or patient acuity measurements. However, the terminology also encompasses concepts like patient classification and nursing workload, which are often used interchangeably [13–15]. In this paper, we will henceforth use the term ‘nursing acuity’ to refer to the workload of nursing staff in both direct and indirect patient-related duties.

It has been demonstrated that accurate acuity measurements can contribute to enhanced patient safety and increased job satisfaction among nurses through a more equitable distribution of workload [13]. It is not given that a patient’s need for nursing care correlates with the severity of their illness [16]. Utilising tools for nursing acuity measurements provides decision support to charge nurses and managers, helping them determine nurse-to-patient ratios and assignments in real time according to patient needs [17].

Various tools and systems with diverse descriptions and definitions for identifying nursing acuity are currently in use. A common limitation among many of these tools is the lack of a validated evidence base [18], and recent literature has not addressed or rectified this gap. Among the validated tools described in the literature, some focus on the general care needs of individual hospitalised patients [18], while others are designed to measure acuity levels for specific specialisations, such as oncology [7] or emergency departments [19]. Nonetheless, a universally accepted standard for measuring nursing acuity within somatic (i.e., related to the physical body rather than mental aspects) in-patient care is currently lacking [16]. Addressing this gap is crucial for accurately assessing nursing acuity levels and resolving inequitable nursing assignments, especially considering that nurse turnover rates are high both in Sweden and internationally [20, 21], which could be attributed to a heavy workload [8]. The impetus for this study stemmed from the need to effectively address nursing acuity in a medium-sized Swedish healthcare region. Consequently, developing and testing a reliable tool through collaborative efforts within the organisation became essential. Therefore, the aim of this study was to describe the development and testing of a tool designed to measure nursing acuity in adult somatic in-patient care.

## Methods and results

This study was reviewed and approved by the Swedish Ethical Review Authority (registration number 2023-03688-01). All procedures adhered to the principles of the 1964 Declaration of Helsinki and its subsequent revisions. This research is part of a larger project, registered with ClinicalTrials.gov (NCT06035705), registration date 7 July 2023.

All statistics analysis were conducted using IBM-SPSS version 29.

### Study setting

The decentralised Swedish public healthcare system is divided into 21 self-governing regions. In the region where this study took place, there are about 285,000 residents and 6,000 employees in the healthcare sector. Like other Swedish regions, there is a shortage of in-patient beds, a complex issue resulting from challenges such as persistent shortages of qualified nursing staff and problems managing patient flows. To address these issues, the top-level healthcare management decided to implement nursing acuity measurements within the organisation. This task was assigned to the authors KD and KM, who work on healthcare development initiatives within the organisation.

From the outset, it was decided that the nursing acuity tool needed to be versatile enough to be used in all adult somatic in-patient wards, thereby facilitating comparisons between wards. Another key need was the ability to distinguish between advanced nursing tasks and basic nursing tasks, based on responsibilities associated with different professions. In Sweden, registered nurses (RN) complete a three-year bachelor’s level university program. Their advanced nursing tasks involve extensive responsibilities, including assessing patient health status, managing medication, planning and evaluating nursing care, collaborating with patients and their relatives, and adapting to changing clinical conditions [22]. In Sweden, the title of assistant nurse (AN) is a protected designation, with education comprising either a three‑year upper secondary program or a 1.5‑year adult education program [23]. In in-patient settings, their tasks often focus on basic nursing care, such as assisting with activities of daily living (ADL), blood sampling, and mobilisation. They work closely with patients and frequently collaborate in teams with RNs. Despite the differing responsibilities, it was decided from the outset not to sharply distinguish between the tasks of RNs and ANs in the acuity tool. This approach aims to avoid creating strong divisions between the professions, support task-shifting, and promote inter-professional teamwork. Furthermore, it was essential to collaborate with the nursing staff throughout the development process. Finally, practical contribution was a high priority, with a focus on developing a tool that is feasible, easy to administer, and straightforward to learn and maintain.

### Tool development

**Fig. 1:**
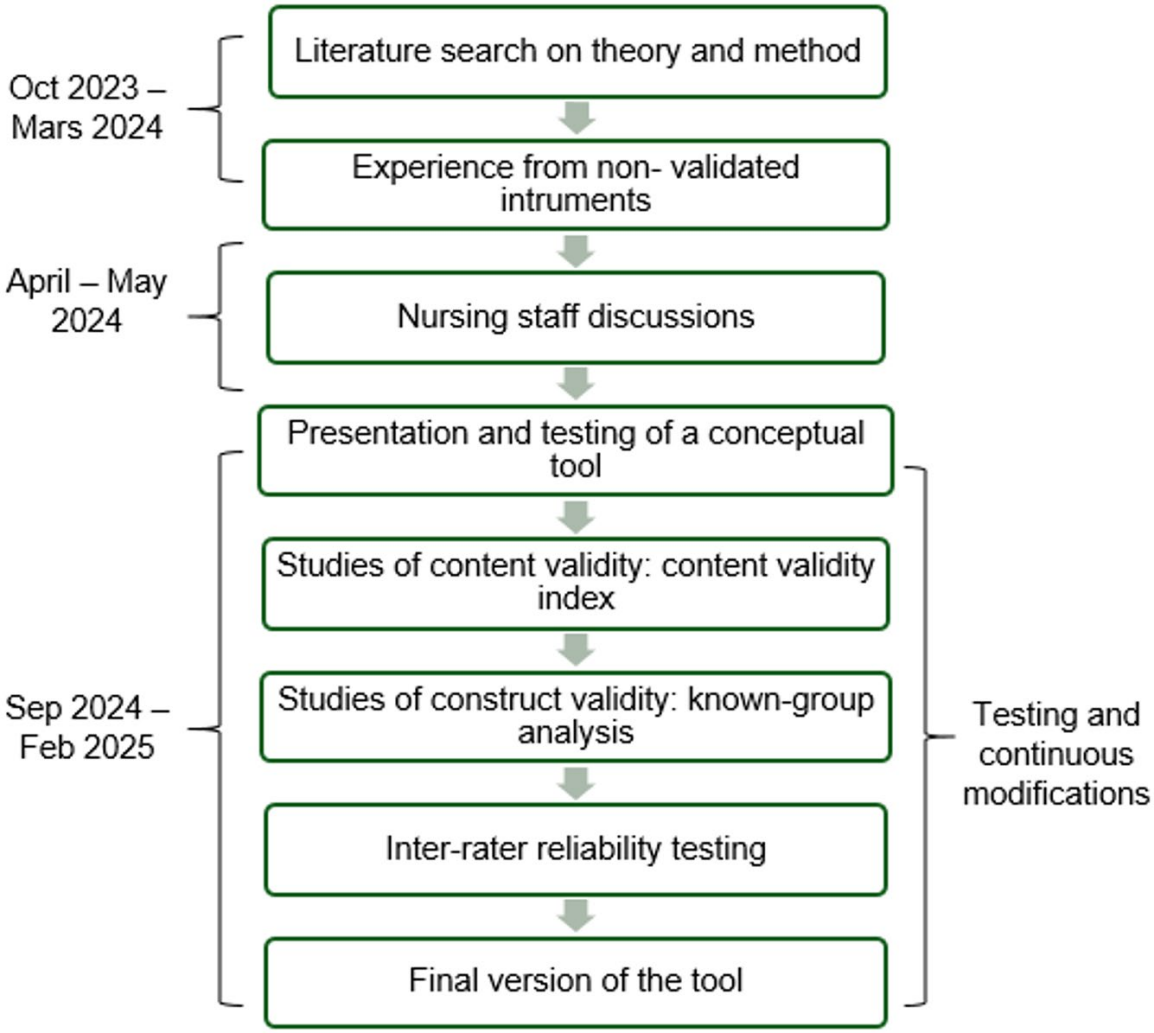
Development process of the nursing acuity tool

The nursing acuity tool was developed through an iterative process in a natural context. The development took place between October 2023 and February 2025. The methodological process for the development, as outlined in Fig. 1 was followed.

### Literature search on theory and method

We examined literature from scientific databases (CINAHL, Ovid Medline, Scopus and Web of Science) to locate empirical and evidence-based studies as well as reviews of empirical research. The search was performed in collaboration with the university library’s search team. A series of key words were identified, and exclusion and inclusion criteria were established [24]. This helped refine the scope of the studies and ensured that only papers relevant to the project’s objectives were reviewed. Some tools found in the literature served as sources of inspiration, such as RAFAELA, a Finish instrument to systematically measure nursing intensity. From this instrument we primarily focused on how they divided the total sum of nursing acuity by the total number of nurses caring for patients in the unit [25]. Another source of inspiration was the Oncology Acuity Tool, which reinforced our conviction that patient-specific, real-time acuity measurements, enabling shift-by-shift staffing adjustments in response to evolving patient care needs, were the right path forward [7].

The literature search revealed no current comprehensive tool, tested for validity and reliability, that was suitable for use in adult somatic in-patient care and supported both daily management and long-term follow-up. Therefore, we needed to develop our own.

### Experience from non-validated instruments

**Fig. 2:**
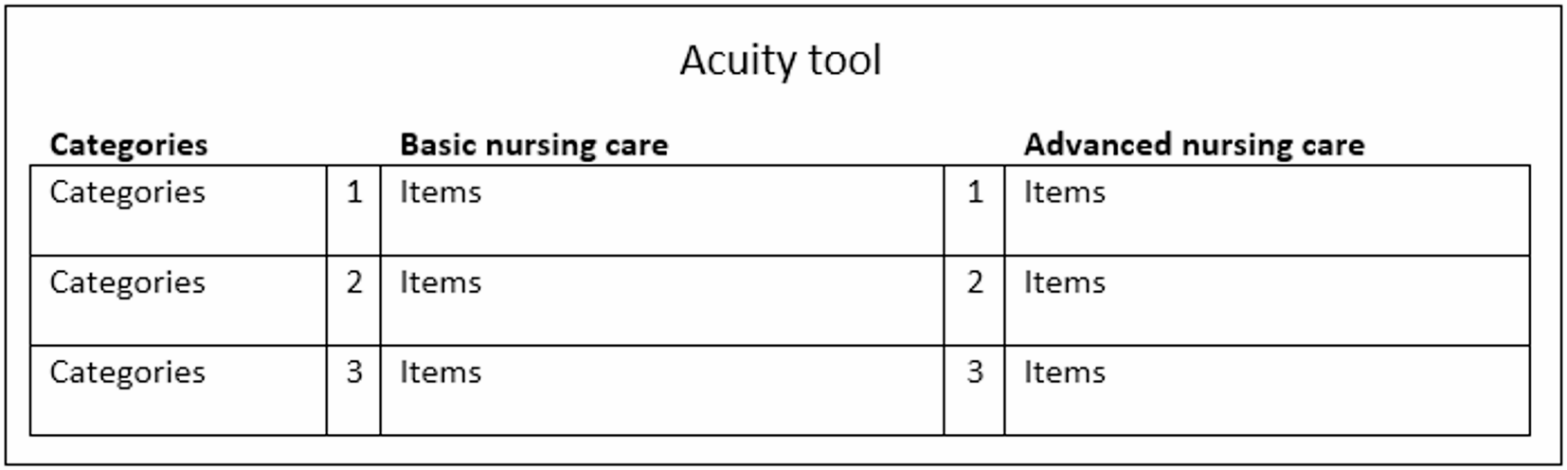
Architecture of the Acuity tool

We drew on experience from three existing non-validated nursing acuity tools currently used in Swedish healthcare settings. These were the only tools that met our criteria for classification into basic and advanced nursing care and were general enough to be applicable across various in-patient care specialties, based on our internet searches and professional contacts. Inspiration for tool design was drawn from a tool used at a major university hospital, designed as a grid model with basic nursing care and advanced nursing care in separate columns, and nursing acuity rated on a scale from 1 to 3 (see Fig. 2). This model was considered easy to overview and had proven useful in clinical practice, which is why this layout was proposed for discussion in the next stage.

The process continued by integrating insights from the literature search and experiences with non-validated instruments to develop a comprehensive list of both direct and indirect patient-related nursing tasks for the subsequent phase. A few items were excluded at this stage due to their lack of relevance to the current healthcare region, being too specific, or already encompassed by other criteria.

### Nursing staff discussions

In this phase, three RNs and three ANs volunteered to participate in the collaborative discussions. On average, the participants, all female, had 11.5 years of work experience within the organisation, with experience ranging from 2 to 36 years (see Table 1).

**Table 1:** Participants in nursing staff discussions

Characteristics	*N*			
Profession				
Registered nurses Assistant nurses	3 3			
Sex				
Male Female	0 6			
Work fields	RNs		ANs	
Orthopaedics Nephrology/haematology Cardiology	1 1 1		1 1 1	
Years in profession Registered nurses Assistant nurses	mean 14 8	median 5 6		range 2–36 5–13

The participants worked at three in-patient wards—orthopaedics, nephrology/haematology, and cardiology—that already addressed nursing acuity in their daily operations. These acuity measurements ranged from simply classifying patients as ‘heavy’ or ‘not heavy’ to rating patients on a self-developed nursing acuity scale from 1 to 7. None of the participating wards conducted any registration or follow-up on their acuity measurements, and only one ward had previously distinguished between basic and advanced care.

Participants met on three occasions, each lasting 90–120 min, for discussions with members of the research group (KD – registered nurse, KM – speech-language therapist, both with experience in organisational development and in-patient care). The starting point of this phase was a broad list of nursing tasks provided to the participants before the first encounter, based on the previous steps in the development process. These tasks were meticulously discussed in terms of relevance and wording. One example of a new item added to the model based on discussions at this stage was ‘significant others in need of substantial support’. Some items were also removed, such as ‘medically ready for discharge’ and ‘protective suit/clothing’, as they were incorporated into broader categories. Certain items important to specific specialties were rephrased to become more generic. Throughout the collaborative discussions, the items were continually refined. Each nursing task was labelled as advanced nursing care or basic nursing care and was rated for nursing acuity on a scale from 1 to 3 based on estimated workload. These tasks were then divided into five overarching categories: care planning, ADL, monitoring/nursing interventions, medication, and cognition. The items were placed at different levels (1–3) based on severity, the amount of time the task requires, the cognitive focus/attention needed from the nursing staff, and the frequency of the interventions per day. When the group had differing opinions about the rating of a task or whether a task belonged to advanced or basic nursing care, a consensus decision was made. Since a few tasks were performed by either ANs or RNs depending on the ward they worked in, emphasis was placed on the fact that the division into basic and advanced nursing care was not fully aligned with professional affiliation. After each meeting, all participants received the updated set of items to ensure informed discussions. During the third and final meeting, the group reached a consensus on a conceptual tool, which served as the foundation for testing and continued development.

### Presentation and testing of a conceptual acuity tool

The conceptual nursing acuity tool was tested at five volunteering somatic in-patient wards within the healthcare organisation. The selection of wards was partly based on specific challenges in recruiting and retaining staff, as well as their willingness to test new ways of working. Additionally, the selection ensured a distribution both geographically and in terms of the area of care. Consequently, these five wards were located across all three hospitals within the region: one medium-sized hospital (Hospital A) and two smaller hospitals (Hospitals B and C), which are geographically dispersed within the region (see Table 2).

**Table 2:** Structure of the test wards

	Hospital	No of beds	No of RN in budget	No of AN in budget	Patient average length of stay (days) Sep 2024 – Feb 2025
Cardiac/cardiac intensive care	C	12	15	13	2,9
Stroke	A	16	20	25	4,8
Medicine/stroke	B	24	30	31	4,4
Orthopaedic	B	18	14	23	4,4
Surgical	A	18	22	21	5,3

Testing of the tool took place between September 2024 and February 2025 and was preceded by rigorous planning of proposed work procedures. Before the test phase began, first- and second-line managers of each ward were provided with detailed information about the process. In this region, managerial responsibilities include accountability for healthcare services provision, budget, work environment, and staffing.

All nursing staff in the participating wards received training on the tool and its application during staff meetings, supplemented by additional information from their first-line manager. The first-line manager played an important role during this phase by encouraging the nursing acuity measurements and establishing conditions for staffing adjustments. At each ward, the conceptual nursing acuity tool was visibly posted on the office wall, next to the whiteboard used for visual control. The tool was also made available electronically, and in pocket-sized format for each nurse. One RN or AN in each ward was appointed nursing acuity coordinator, responsible for both materials and work related to the acuity measurements within the staff group. Due to time constraints among RN, it was more common for AN to take on the role of nursing acuity coordinator. The patient beds within all five test wards are divided into several ‘care teams’, each staffed by at least one RN and one AN, depending on the number of patients and the specialty. The nursing acuity measurement processes were delineated as follows: after each day and evening shift, nursing acuity was collaboratively assessed by the care team staff for each of their patients (about 3 to 6) at each care team. The time required was approximately 3–5 min per team, depending on the number of patients. The assessment tool placed in front of the whiteboard, used to get an overview of all patients on the ward and their patient-responsible nursing staff (visual control). As a minimum, at least one RN and one AN from each care team should participate. Although these acuity measurements were based on the conceptual tool, joint discussions were essential, as several items in the tool also relied on subjectivity. Reporting of nursing acuity was done directly on the whiteboard using coloured magnets that indicated nursing acuity for basic nursing care and advanced nursing care. To oversee the nursing acuity measurements from a broader perspective, the measurements were also documented on an iPad and stored in a database.

During the first weeks of the test phase, KD and KM visited each ward, observed the measurements and requested feedback on the tool. Additionally, monthly meetings were held with all first-line managers and nursing acuity coordinators. During these meetings, the tool and its usage were discussed, and participants proposed adjustments and provided feedback from their colleagues. At this stage, some staff suggested that using ratings based on a 5-point scale would have been beneficial for certain items in the tool. However, the participating wards considered it preferable to keep the 3-point scale, as it was simpler to overview. The most significant adjustment during the test period was the reordering of the tool, with the criteria for the highest nursing acuity of 3 placed at the top. The rationale behind this change was that once a patient meets a criterion for 3, the remaining parts of the tool do not need to be reviewed. Additionally, some items were added, such as ‘Telemetry surveillance indicating the need for subacute medical intervention,’ while others were adjusted due to wording issues.

### Studies of content validity index (CVI)

The item-level CVI (I-CVI) was used to calculate the content validity index for the items in the nursing acuity tool. To assess CVI, a panel of experts consisting of ten nursing staff from the test wards was contacted using purposive sampling. The inclusion criteria for participation in the expert group were that the participant should hold a permanent position and have worked with the nursing acuity measurements during the test period. For an overview of participating experts, see Table 3.

**Table 3:** Content validity panel

Characteristics	*N*			
Profession				
Registered nurses Assistant nurses	4 6			
Sex				
Male Female	3 7			
Work fields Orthopaedics Internal medicine/stroke Stroke Surgery Cardiology	RNs 1 1 1 0 1		ANs 1 1 1 2 1	
Years in profession Registered nurses Assistant nurses	mean 3.25 15.7	median 4 13.5		range 1–4 5–25

The participants were asked to evaluate each tool item based on its relevance to the underlying construct, using a 4-point scale, where 1 = not relevant, 2 = somewhat relevant, 3 = quite relevant, 4 = highly relevant [26]. Additionally, they were encouraged to provide comments, with feedback being mandatory for ratings of 1 or 2. For each item, the I-CVI was then computed as the number of experts giving a rating of either 3 or 4, divided by the number of experts. An I-CVI of 0.80 or higher is considered acceptable [27], and all 70 items met this criterion. Twenty-nine items with values below 1.0 are included in Supplementary Material 1. The overall S-CVI for the conceptual nursing acuity tool was then determined by averaging the I-CVI values across all items, resulting in an S-CVI of 0.95. This provides robust evidence of the tool’s content validity, with 0.80 as the lower limit of acceptability for an S-CVI [27]. All comments from the participants were carefully considered, and minor revisions to improve clarity were made.

### Studies of construct validity using known-group analysis

Known-groups validity tests hypotheses about a measure’s ability to distinguish between two or more groups that are known to differ on the construct of interest [26]. A known-group analysis was conducted to evaluate the construct validity of the nursing acuity tool by comparing the tool outcomes between different hospital wards, based on hypothesised (H) relationships.

H1: Patients in the combined medicine/stroke unit at Hospital B are expected to have higher nursing acuity outcomes for basic nursing care than patients in the cardiac/cardiac intensive care ward at Hospital C. This assumption is based on the nature of the patients treated in these wards, with many patients in the medicine/stroke unit having disabilities and multiple chronic conditions, while patients in the cardiac/cardiac intensive care ward tend to be younger and more ambulatory.

H2: There will be no difference in nursing acuity outcomes for basic nursing care between patients in the stroke ward at Hospital A and those in the stroke unit at the stroke/medicine ward at Hospital B, given the similar patient composition.

H3: Patients in the cardiac/cardiac intensive care ward at Hospital C are expected to have significantly higher nursing acuity outcomes for advanced nursing care than patients in the orthopaedic ward at Hospital B. This is due to the advanced nursing responsibilities involved in intensive cardiology [28], whereas the orthopaedic ward primarily consists of individuals receiving postoperative care following orthopaedic procedures.

For the present analysis, 500 nursing acuity measurements per ward, collected between September and December 2024, were randomly selected. The data, originally entered by care teams via ward-specific iPad interfaces, were exported from R into Excel, where the program’s random selection function was used to generate the sample. Independent samples t-tests and Cohen’s d (for effect size) were used to examine whether the nursing acuity outcomes could distinguish between wards with different patient groups. Prior to conducting the t-tests, visual inspection of histograms indicated that the nursing acuity outcome data for each group were approximately normally distributed.

In line with H1, there were statistically significantly higher means for basic nursing care at the medicine/stroke ward (t = -6.99, *p* < 0.001, Cohen’s d = -0.442). H2 was also supported; nursing acuity outcomes for basic nursing care did not differ significantly between the stroke ward at Hospital A and the stroke unit at the medicine ward at Hospital B (t = 0.85 *p* = 0.396, Cohen’s d = 0.054). Additionally, the mean difference in nursing acuity for advanced nursing care was statistically significantly higher at the cardiac/cardiac intensive care ward at Hospital C than at the orthopaedic ward at Hospital B (t = 9.96, *p* < 0.001, Cohen’s d = 0.630). These findings reinforce the ability of the nursing acuity tool to effectively differentiate between varying levels of care needs across different hospital wards, thereby supporting its construct validity.

### Inter-rater reliability (IRR)

IRR was assessed by nursing staff using fictitious cases loosely based on real cases encountered during on-site observations from the participating wards. Twenty-five cases were written by KD, reviewed by KM, ME and AB, and subsequently authenticated by an independent expert from each specialty. The case format was used to ensure that each rater had access to the same information. This procedure has previously been used in IRR assessments, in relation to acuity evaluations [7].

The same 10 nursing staff members previously enrolled in the content validity panel (see Table 3) were asked to individually rate the acuity level of all 25 written cases based on the nursing acuity tool, without any time constraints. They were instructed to base their ratings solely on the written information provided and to use the same approach they employ to rate acuity in their ward. IRR was computed for both the basic nursing care and the advanced nursing care categories, assessed using the intraclass correlation coefficient (ICC) [26] in IBM-SPSS version 29. Two-way random effects model was used where both people and measures effects are random, and an absolute agreement (type A intraclass correlation coefficient). The ICC, average measures, for basic nursing care was 0.955, and for advanced nursing care, it was 0.905. Values above 0.9 are considered to indicate excellent reliability [29].

### Final version of the tool

The iterative process of development and testing described above culminated in the final version of a tool for nursing acuity measurements for adult somatic in-patient care, subsequently named the Swedish Nursing Acuity Measurement (NAM-S). Results indicate acceptable validity and reliability. The NAM-S is presented in Supplementary Material 2 and is divided into two domains: basic nursing care, which includes fundamental nursing activities, and advanced nursing care, which encompasses more complex and specialised nursing tasks. Each domain is assessed on a scale with a set of predefined items, a total of 67 items, (37 for basic nursing care and 30 for advanced nursing care). These items are divided into five categories: care planning, ADL, monitoring/nursing intervention, medication, and cognition, ranging from 3 to 1, where 3 represents the highest level of nursing acuity.

## Discussion

The present study provides information about the development and the psychometric robustness of the NAM-S, a tool for measuring nursing acuity across different specialities. The tool is designed for practical use by nursing staff and managers in adult somatic in-patient care. To our knowledge, this is the first validated instrument developed specifically for this setting. Our results indicate acceptable validity, including content and construct validity, as well as acceptable inter-rater reliability. Although these indications of the psychometric features of the NAM-S are positive, additional work is required to determine its effectiveness as a tool for nursing acuity measurements used as a foundation for determining nurse-to-patient ratios and assignments according to patient needs.

When developing the NAM-S, one of the primary goals was to create a comprehensive tool that does not add to the already considerable workload of nursing staff. From the outset, NAM-S was co-created with its intended users, i.e., the nursing staff—and pilot-tested over a six-month period in five different in-patent wards before validity testing took place. Staff members contributed actively to the discussions, and all test wards demonstrated sustained engagement and interest throughout the development process.

Continuous modifications were made throughout the process, including notable changes, such as reordering the rating scale. These iterative adjustments might explain the demonstrated acceptable validity and inter-rater reliability, as well as the more modest modifications made to the tool during the final step of the development process.

In their study, Brennan et al., [7] discuss the methodological challenges involved in validating a nursing acuity tool for making nurse assignment and staffing decisions in in-patient oncology care. The authors suggest forming clinical-research partnerships and balancing traditional statistical guidelines with clinical relevance when validating measures closely tied to clinical processes. By employing a highly collaborative process to ensure authenticity and relevance, our intention was to address these methodological challenges.

It must be acknowledged that NAM-S includes several items that contain a certain degree of subjectivity and can be considered more vague indicators, such as anxiety, high need for social interaction, or family member requiring support from the staff. However, the inclusion of these items is also a strength of the tool, as it recognises that a patient’s need of nursing care cannot always be directly equated to the severity of their illness [16]. Nursing care is multifaceted and complex, and a nursing acuity tool that incorporates subjective items allows for a more comprehensive and nuanced understanding. For instance, subjective factors as previously described, are difficult or impossible to quantify in the electric health record (EHR). Through testing of the instrument’s validity and reliability, we have addressed the potential issues related to subjectivity. Our validation process ensures that the tool accurately measures what it is intended to measure, and our reliability testing confirms that it produces consistent results across different users. Nevertheless, it is important to recognise that certain items may still be interpreted differently by two different nurses.

The psychometric robustness of the NAM-S does not guarantee that the nursing acuity measurements will automatically be accurate and dependable when the NAM-S is utilised. It is crucial that assessments are conducted by staff familiar with the patient, and that evaluations are carried out collaboratively by RNs and ANs, as effective teamwork has previously been demonstrated to be central to patient safety [30]. Thorough introduction and ongoing education about assessments using the NAM-S are essential in order to avoid substandard ratings, and a concise manual on its use is currently being developed.

It has previously been discussed that a limitation of acuity tools is that they rarely account for differences in capacity between nurses [31]. Similarly, differences in work experience among nurses might lead to variations in estimated workload due to differences in the time and effort required to complete a task. Consideration must also be given to the fact that the NAM-S encompasses only direct and indirect nursing tasks related to individual patients. Additionally, there are also several indirect nursing assignments, not connected to a specific patient, such as material administration, equipment preparation, and mealtime preparation, which significantly impact workload but are not included in the tool. The extent of these tasks may vary between wards and must be acknowledged when discussing nursing acuity, especially in comparisons between different wards.

This study has considerable strengths. One illustrative example is the continuous dialogue conducted between ANs, RNs, managers, and the research team throughout the development process. These interactions were essential to ensure the tool’s usability for professionals integrating it into routine clinical practice. Another notable strength is that the tool has been evaluated across multiple disciplines, thereby supporting its comparability and applicability within both medical and surgical specialties.

Despite its strengths, it can also be noted as a limitation in the development and testing of the NAM-S that it was not evaluated across all specialities within somatic in-patient care where the tool is intended to be used. In the organisation where the tool was developed, only a medium-sized and two small hospitals were represented. Consequently, there may be patient groups and more complex health conditions whose needs are not addressed by the NAM-S items. It would be beneficial to further examine the NAM-S in connection with a broader implementation or in other healthcare organisations, including large-sized or university hospitals, and ultimately, in healthcare settings in countries outside Sweden. It most also be noted as a limitation that the expert group comprised four RNs and six ANs. Another limitation in the development process that the weights of the tool items on the 1–3 scale were based solely on professional experience and consensus, rather than real-time assessments of nursing task durations. Additionally, the inter-rater reliability testing procedure was conducted individually by RNs and ANs rather than through consensus discussions. This approach deviates from the intended use of the tool, which relies on collaborative evaluations to ensure accuracy and consistency in real-life clinical practice.

Something that could be noted as both a strength and a limitation is that two of the authors (KD, KM) were responsible for the work on nursing acuity measurements within the organisation while simultaneously taking on the roles of researchers. Their pre-existing knowledge of the organisation and its culture may have been a hindering factor in perceiving things critically [32], but it can also provide a deeper understanding and be crucial for interpreting results in light of previous research [26].

Nurse staffing is essential for ensuring patient safety and improving outcomes. In theory, the concept is simple: match the number of nurses available to the needs of the patients. However, in practice, maintaining this balance is quite challenging. Using the NAM-S as a basis for daily staffing decisions and adjustments may help achieve this balance within somatic adult in-patient care. It must be acknowledged that the tool is not a solution in and of itself. To properly address nursing acuity, tasks such as assignment planning and staff allocation require effective managerial decisions [33]. Without such activities based on NAM-S ratings, it is unlikely that benefits such as a more equitable workload will be achieved.

## Conclusions

In this paper, a tool for measuring nursing acuity (NAM-S) in adult somatic in-patient care has been developed and tested. The NAM-S demonstrated both validity and reliability and is considered ready for evaluation in larger-scale settings. To further refine the model, additional examinations of its usefulness and experiences regarding the practical use of NAM-S is warranted.

## Supplementary Information

Below is the link to the electronic supplementary material.

Supplementary Material 1

Supplementary Material 2

Supplementary Material 3

## Data Availability

The datasets used and analysed during the current study are available from the corresponding author upon reasonable request. Signing a data sharing agreement will be necessary. Individual data are unavailable due to General Data Protection Regulations (GDPR) and in accordance with the ethics application.

## Abbreviations

NAM-S: Nursing Acuity Measurement–Swedish version
RN: Registered nurse
AN: Assistant nurse
EHR: Electronic health record
