# Supplementary material for: Tool for Nursing Acuity Measurement - Swedish version (NAM-S) for somatic in-patient care: development, validity, and reliability
Source: BMC Health Serv Res. 2026 Jan 27;26:274. doi: 10.1186/s12913-026-14036-w (PMC12918532; doi:10.1186/s12913-026-14036-w)
Supplement: Supplementary file 1 — Supplementary Material 1 [file 12913_2026_14036_MOESM1_ESM.docx]

**Content validation summary, comments, and revisions of the checklist**

| **Basic Nursing Care** | **Item** | **I-CVI** | **Recommendations (mandatory för score >2) or comments** | **Revisions to checklist** |
| --- | --- | --- | --- | --- |
| 3 | Nausea (caused by medication) | .90 | “If the patient manages nausea and vomiting on their own and only vomits once in a bag, it may not really affect the workload for the nursing staff.” | Removed: item removed due to uncertainty about how much it may come to affect the workload. |
|  | Total parenteral nutrition (TPN) | .90 | "Switch to advanced nursing care. It's important to keep an eye on the insertion site because there's a risk that the infusion could go into the subcutaneous tissue, which can cause thrombophlebitis." | Moved to Advanced Nursing Care: this is primarily within the professional domain of RN. |
|  | Blood sampling 4 times per day or more | .90 | “Does this refer to venous or capillary sampling?" | Clarified: Sampling/b-glucose (venous or capillary) at 4 times/day or more. |
| 2 | Day of admission or discharge | .80 | "The workload on admission and discharge days depends on the specific needs of each patient."  “Move to Advanced Nursing Care” | Clarified: Time-consuming activities during admission och discharge. Moved to Advanced Nursing Care. |
|  | Supportive measures (e.g., need for an interpreter) | .90 | "Why not combine this with other communication difficulties, such as aphasia?” | Moved to Communication difficulties and rephrased to Communication difficulties due to language or aphasia. |
|  | Drain care maximum 3 times a day | .89 | No recommendations or comments. | No action |
|  | Basic care planning via LifeCare (*communication systems between healthcare providers*) (carried out by nursing staff) | .90 | "In the absence of a designated care planner, the responsibility for this task falls to the registered nurses." | Moved to Advanced Nursing Care.  The term 'via LifeCare' was removed in order to enhance the general applicability of the tool, and has been replaced with the designation 'Basic care planning conducted by nursing staff'. |
|  | Outplaced patient | .89 | No recommendations or comments. | No action |
|  | Family member in need of support from a nursing assistant | .90 | No recommendations or comments. | No action |
| 1 | Elective patient | .89 | "I don't understand what the word 'elective' means." | Clarified: Elective patient (planned admission for surgery or treatment). |
|  | The patient requires no assistance with mobilisation and can be mobilised on the same day following surgery | .90 | No recommendations or comments. | Removed: item that do not contribute any value to the measurement of nursing acuity. |
|  | Urine and stool normal | .89 | "If it is considered normal, then why should it be included?" | Removed: items that do not contribute any value to the measurement of nursing acuity. |
|  | No side effects from morphine preparations, (such as nausea or confusion) | .90 | "Why morphine specifically? All medications can have side effects..." | Removed: incorporated within other items. |
|  | Anxiety is managed largely independently | .90 | No recommendations or comments. | No action |

| **Advanced Nursing Care** | **Item** | **I-CVI** | **Recommendations (mandatory för score >2) or comments** | **Revisions to checklist** |
| --- | --- | --- | --- | --- |
| 3 | Coordinated Individual Plan | .90 | No recommendations or comments. | No action |
|  | Booking of transport for out-of-area patient | .80 | No recommendations or comments. | No action |
|  | Continuous Positive Airway Pressure (CPAP) | .90 | No recommendations or comments. | No action |
|  | Tablets 7 times a day or more | .90 | No recommendations or comments. | No action |
| 2 | Percutaneous endoscopic gastrostomy (PEG)/feeding tube | .90 | No recommendations or comments. | Moved to Care |
|  | Care of nasopharyngeal catheter | .90 | No recommendations or comments. | No action |
|  | Telemetry monitoring assessed by RN | .90 | No recommendations or comments. | No action |
|  | Blood transfusions | .90 | No recommendations or comments. | No action |
|  | Insulin-treated diabetes | .90 | No recommendations or comments. | No action |
|  | Tablets maximum 6 times a day | .90 | No recommendations or comments. | No action |
|  | Injection/inhalation as needed | .90 | No recommendations or comments. | No action |
|  | Central dialysis catheter | .90 | No recommendations or comments. | No action |
| 1 | Tablets maximum 4 times a day | .90 | No recommendations or comments. | No action |
|  | Injection/inhalation in conjunction with other medication administration | .90 | No recommendations or comments. | No action |

Only items with a value below 1.0 are included in the table, representing 29 out of 70 items. I-CVI of .80 or higher is however considered acceptable^[[1]](#footnote-1)^, and all 70 items met this criterion. For the items that received the comment 'No action', the research group has assessed that these still contribute value in the context of measuring nursing acuity.

1. Polit DF, Beck CT. Nursing Research Generating and Assessing Evidence for Nursing Practice. Vol. 2021. [↑](#footnote-ref-1)
