# Supplementary material for: Tool for Nursing Acuity Measurement - Swedish version (NAM-S) for somatic in-patient care: development, validity, and reliability
Source: BMC Health Serv Res. 2026 Jan 27;26:274. doi: 10.1186/s12913-026-14036-w (PMC12918532; doi:10.1186/s12913-026-14036-w)
Supplement: Supplementary file 2 — Supplementary Material 2 [file 12913_2026_14036_MOESM2_ESM.docx]

**Nursing Acuity Measurement – Swedish version (NAM-S)**

To be classified at a higher level, it is sufficient that one item is fulfilled.

|  | **Categories** |  | **Basic nursing care** |  | **Advanced nursing care** |
| --- | --- | --- | --- | --- | --- |
|  | Care Planning | **3** | Need for close observation. | **3** | Coordinated Individual Care Plan or more complex care planning (conducted by care staff), or enrollment in a palliative care team. Booking of transport for out-of-area patients. |
|  | Activities of daily living (ADL) |  | Assistance required with all activities of daily living (ADL). Mobilisation requires lifting by 2–3 staff members. |  |  |
|  | Monitoring/  Nursing  interventions |  | Nation Early Warning Score (NEWS) observation > 6 times per day. Blood sampling/blood glucose (venous or capillary) at 4 or more times per day. Frequent repositioning/turning schedule. Continuous bladder irrigation (CBI) with complications, e.g., manual flushing twice per hour. Tracheostomy care. Drainage and central venous access in a patient with motor agitation. Drain care more than 3 times per day. |  | Arrhythmia/ischemia requiring urgent intervention. Continuous Positive Airway Pressure (CPAP)/ Optiflow/ Bilevel Positive Airway Pressure (BiPAP). Total parenteral nutrition (TPN). Time-consuming or complex wound dressing requiring multiple staff members. |
|  | Medication |  |  |  | Insulin-treated diabetes with unstable blood glucose levels. Tablets 7 times per day or more. Medications requiring additional monitoring during administration. Advanced pharmacological treatment such as chemotherapy. |
|  | Cognition |  | Anxiety. Confusion. Dementia/cognitive impairment. High need for social interaction. |  |  |
|  | Care Planning | **2** | Outplaced patient requiring additional nursing interventions. | **2** | Time-consuming activities during admission or discharge. Basic care planning conducted by nursing staff). |
|  | Activities of daily living (ADL) |  | Assistance is required with activities of daily living (ADL), such as personal hygiene, feeding, and dressing. Mobilisation with the help of one staff member. Risk of falling. |  |  |
|  | Monitoring/  Nursing  interventions |  | Nation Early Warning Score (NEWS) observation 3–5 times per day. Blood sampling/blood glucose (venous or capillary) 2–3 times per day. Difficult venous access. Continuous infusion/urine output monitoring (hourly diuresis). Barrier nursing/increased use of protective equipment/isolation precautions. Drain care up to 3 times per day. Rehabilitation or training prescribed by another professional category (e.g., paramedical staff). Percutaneous endoscopic gastrostomy (PEG) or tube feeding." |  | Daily wound dressings. Midline catheter (long-term peripheral venous catheter). Central venous catheter (CVC). Dialysis managed by ward staff. Care of nasopharyngeal catheter. Telemetry monitoring assessed by registered nurse. Arrhythmia requiring subacute intervention. |
|  | Medication |  |  |  | Blood transfusions. Insulin-treated diabetes. Tablets up to 6 times per day. Injection/inhalation as needed. Epidural Analgesia (EDA)/ Patient-Controlled Analgesia (PCA). Adverse effects requiring intervention. |
|  | Cognition |  | Mild anxiety/confusion. Communication difficulties due to language barriers or aphasia. Family member requiring support from assistant nurse. |  | Family member requiring support from registered nurse. |
|  | Care Planning | **1** | Elective patient (planned admission for surgery or treatment). | **1** |  |
|  | Activities of daily living (ADL) |  | Manages activities of daily living (ADL) independently or requires minimal assistance. |  |  |
|  | Monitoring/  Nursing  interventions |  | Nation Early Warning Score (NEWS) observation 1–2 times per day. Blood sampling/blood glucose (venous or capillary) up to once per day. |  |  |
|  | Medication |  |  |  | Intravenous fluids, e.g., glucose or Ringer’s solution. Tablets up to 4 times per day. Injections/inhalations administered concurrently with other medication rounds. |
|  | Cognition |  | Anxiety mainly managed independently. |  |  |
