# Supplementary material for: Tool for Nursing Acuity Measurement - Swedish version (NAM-S) for somatic in-patient care: development, validity, and reliability
Source: BMC Health Serv Res. 2026 Jan 27;26:274. doi: 10.1186/s12913-026-14036-w (PMC12918532; doi:10.1186/s12913-026-14036-w)
Supplement: Supplementary file 3 — Supplementary Material 3 [file 12913_2026_14036_MOESM3_ESM.docx]

**Supplementary files:** summary of the measurement results and t-test assumptions

| **Independent Samples T-test** | | | | | | |
| --- | --- | --- | --- | --- | --- | --- |
|  |  | **Statistic** | **df** | **p** |  | **Effect Size** |
| H1 | Students´s t | -6.99^a^ | 998 | < .001 | Cohen´s d | -0.442 |
|  | Welch´s t^b^ | -6.99 | 957 | < .001 |  |  |
|  |  |  |  |  |  |  |
| H2 | Student´s t | 0.848 | 998 | 0.396 | Cohen´s d | 0.0537 |
|  |  |  |  |  |  |  |
| H3 | Students´s t | 9.96^a^ | 998 | < .001 | Cohen´s d | 0.630 |
|  | Welch´s t^b^ | 9.96 | 928 | < .001 |  |  |

^a^ Levene´s test is significant (p < .05)

^b^ Levene’s test was significant in two of the analyses (H1, H3); accordingly, Welch’s test was applied in those cases.

| **Assumptions (Homogeneity of Variances Test (Levene´s)** | | | | | | |
| --- | --- | --- | --- | --- | --- | --- |
|  | **F** | **df** | **df2** | **p** |  |  |
| H1 | 37.0 | 1 | 998 | < .001 |  |  |
|  |  |  |  |  |  |  |
| H3 | 207 | 1 | 998 | < .001 |  |  |
|  |  |  |  |  |  |  |

Visual check of histograms indicated a normal distribution.
